# Settling Down: The Genome of *Serratia symbiotica* from the Aphid *Cinara tujafilina* Zooms in on the Process of Accommodation to a Cooperative Intracellular Life

**DOI:** 10.1093/gbe/evu133

**Published:** 2014-06-19

**Authors:** Alejandro Manzano-Marín, Amparo Latorre

**Affiliations:** ^1^Institut Cavanilles de Biodiversitat i Biologia Evolutiva, Universitat de València, Spain; ^2^Unidad Mixta de Investigación en Genómica y Salud, Centro Superior de Investigación en Salud Pública, Valencia, Spain

**Keywords:** *Serratia symbiotica*, *Buchnera aphidicola*, co-obligate, aphid endosymbiont, genome reduction, riboflavin

## Abstract

Particularly interesting cases of mutualistic endosymbioses come from the establishment of co-obligate associations of more than one species of endosymbiotic bacteria. Throughout symbiotic accommodation from a free-living bacterium, passing through a facultative stage and ending as an obligate intracellular one, the symbiont experiences massive genomic losses and phenotypic adjustments. Here, we scrutinized the changes in the coevolution of *Serratia symbiotica* and *Buchnera aphidicola* endosymbionts in aphids, paying particular attention to the transformations undergone by *S. symbiotica* to become an obligate endosymbiont. Although it is already known that *S. symbiotica* is facultative in *Acyrthosiphon pisum*, in *Cinara cedri* it has established a co-obligate endosymbiotic consortium along with *B. aphidicola* to fulfill the aphid’s nutritional requirements. The state of this association in *C. tujafilina*, an aphid belonging to the same subfamily (Lachninae) that *C. cedri*, remained unknown. Here, we report the genome of *S. symbiotica* strain SCt-VLC from the aphid *C. tujafilina*. While being phylogenetically and genomically very closely related to the facultative endosymbiont *S. symbiotica* from the aphid *A. pisum*, it shows a variety of metabolic, genetic, and architectural features, which point toward this endosymbiont being one step closer to an obligate intracellular one. We also describe in depth the process of genome rearrangements suffered by *S. symbiotica* and the role mobile elements play in gene inactivations. Finally, we postulate the supply to the host of the essential riboflavin (vitamin B_2_) as key to the establishment of *S. symbiotica* as a co-obligate endosymbiont in the aphids belonging to the subfamily Lachninane.

## Introduction

Symbiosis between bacteria and insects is a widespread phenomenon that is considered the key to the ability of this group of organisms to occupy a huge variety of niches (Moya et al. 2008). Specifically, many insects sustain mutualistic interactions with a variety of intracellular endosymbiotic bacteria (Kikuchi 2009). Most of these associations have been proven to have a metabolic foundation, where the symbiont provides nutrients that are lacking from the host’s diet. This has been established mostly by genomic (Wu et al. 2006; McCutcheon and Moran 2007; Kirkness et al. 2010; Penz et al. 2010; Shigenobu and Wilson 2011; Sloan and Moran 2012; Tokuda et al. 2013), as well as transcriptomic (Hansen and Moran 2011), proteomic (Poliakov et al. 2011; Fan et al. 2013), and in vivo experimental studies (Moran, Dunbar, et al. 2005; Russell et al. 2013).

Among these types of insect–bacteria consortia, the symbiotic relationship that most aphids maintain with their obligate intracellular bacterium *Buchnera aphidicola* is probably the best studied case, with genome sequences from seven different aphid host species currently available (Shigenobu et al. 2000; Tamas et al. 2002; van Ham et al. 2003; Perez-Brocal et al. 2006; Moran et al. 2009; Degnan et al. 2011; Lamelas, Gosalbes, et al. 2011; MacDonald et al. 2011). *Buchnera aphidicola* is vertically transmitted, confined to bacteriocytes (specialized host cells developmentally determined independently of the bacteria [Braendle et al. 2003]), and has been coevolving with its aphid partner for millions of years (Moran et al. 1993). The endosymbiont provides, in cooperation with the aphid host, essential amino acids (EAAs) required by this, because its phloem-restricted diet is rich in carbohydrates but poor in EAA (Hansen and Moran 2011). *Buchnera* displays extensive genome degradation (van Ham et al. 2003) and high genome stability (Tamas et al. 2002). This has been explained by an initial massive loss of genes (promoted by its newly acquired lifestyle in a safe and rich environment, which in turn lowered the selective pressure on many disposable genes) followed by cospeciation with its respective aphid host (van Ham et al. 2003). Thus, this ancient obligate endosymbiont displays convergent characteristics of many other ancient insect endosymbionts, such as high coding density, low G+C content, absence of mobile genetic elements, and depleted DNA recombination and repair pathways (McCutcheon and Moran 2012). *Buchnera aphidicola* from *Cinara cedri* (hereafter BCc) and *C. tujafilina* (hereafter BCt), both of which belong to the Lachninae subfamily, Eulachnini tribe, possess the smallest *Buchnera* genomes recorded to date (Perez-Brocal et al. 2006; Lamelas, Gosalbes, et al. 2011) (supplementary table S1, Supplementary Material online).

Along with *B. aphidicola*, many aphids can also harbor a variety of other “more recently” acquired secondary endosymbionts, such as *Regiella insecticola* (Degnan et al. 2009), *Hamiltonella defensa* (Degnan, Yu, et al. 2009), *Rickettsia* (Sakurai et al. 2005), *Rickettsiella viridis* (Tsuchida et al. 2014), *Wolbachia* (Gómez-Valero et al. 2004), and *Serratia symbiotica* (Moran, Russell, et al. 2005). These secondary endosymbionts generally display characteristics contrasting those of ancient obligate (also called primary) endosymbionts, including a lower coding density, presence of mobile genetic elements, larger genome sizes, a higher G+C content, and a high number of pseudogenes (Degnan et al. 2009; Newton and Bordenstein 2011; Oakeson et al. 2014). These endosymbionts can establish facultative associations, meaning that the bacteria are not necessary for the partners survival, or co-obligate ones, forming a tripartite symbiotic system with the aphid and its already established obligate symbiont.

The role of secondary endosymbionts may not only be limited to a nutritional one, as is usually the case for primary ones, but it can range from acting as defensive symbionts against parasitoid wasps (Oliver et al. 2003; Hansen et al. 2012), fungal parasites (Scarborough et al. 2005), relating to plant utilization (Henry et al. 2013), and to resistance after heat stress in the form of reproductive advantage. This last trait has been attributed to the facultative endosymbiont *S. symbiotica* in the pea aphid *Acyrthosiphon pisum* (Montllor et al. 2002; Burke et al. 2010). In addition, it was also found to be able to restore the survival and partially the reproduction of *Buchnera*-free aphids while negatively affecting the growth and number of offspring in *A. pisum* (Koga et al. 2003).

Members of the genus *Serratia* can be found on a variety of environments such as water, soil, plants, humans, and invertebrates (Grimont and Grimont 2006). The endosymbiotic *S. symbiotica* present in different aphid species, diverge into two phylogenetic clades (according to a reconstruction using the 16 rRNA gene), clusters A and B (Lamelas et al. 2008). Moreover, *S. symbiotica* representatives from each clade show different cell shapes, sizes, and locations inside the aphid host (Moran, Russell, et al. 2005; Lamelas et al. 2008). In the pea aphid *A. pisum*, *S. symbiotica* (cluster A) presents characteristics of a typical facultative endosymbiont, because it is dependent on *B. aphidicola* for nutrient provisioning, whereas this is not on *S. symbiotica* (Burke and Moran 2011) and is located in sheath cells, secondary bacteriocytes, and haemocel (Moran, Russell, et al. 2005). On the other hand, in the aphid *C. cedri*, *S. symbiotica* (cluster B) is restricted to bacteriocytes, has a spherical cell shape (Lamelas et al. 2008) and it has been determined to be a co-obligate endosymbiont of *B. aphidicola* (Lamelas et al. 2011). So far, this is the only known case where *S. symbiotica* is needed to cooperate with *B. aphidicola* in order to synthesize the EAA tryptophan (Gosalbes et al. 2008).

The aphid *C. tujafilina*, apart from harboring the obligate endosymbiont *B. aphidicola*, has been also found to house *S. symbiotica* (Russell et al. 2003; Lamelas et al. 2008). Surprisingly, this *S. symbiotica* endosymbiont is not closely related to that of the host’s close relative *C. cedri*, as it belongs to the other phylogenetic clade (cluster A) within the endosymbiotic *Serratia*. Additionally, both *S. symbiotica* differ in shape, size, and location inside the host, being in *C. tujafilina* rod shaped and located in sheath cells, secondary bacteriocytes, and extracellularly (Lamelas et al. 2008), as in *A. pisum* (Moran, Russell, et al. 2005; Fukatsu et al. 2000). The differences between both *S. symbiotica* from *C. cedri* and *C. tujafilina* become even more striking when considering the similarity of *B.aphidicola* endosymbionts from both hosts. They have both suffered a massive common gene loss affecting mainly the metabolism of biotin, glutathione, pyrimidines, the electron transport chain, and riboflavin, rendering them functionally very similar (Lamelas, Gosalbes et al. 2011).

In the present work, we have sequenced the genome of *S. symbiotica* from the aphid *C. tujafilina* and compared it with the genome of both *S. symbiotica* from *A. pisum* and *C. cedri*, as well as with the ones of free-living *Serratia*. We have been able to determine its phylogenetic positioning and elucidate the process of genome reduction undergone, not only by *S. symbiotica* from *C. tujafilina* but also by the other *S. symbiotica*. We also describe the genome reordering, gene inactivation, and the role that mobile elements have played in these processes. Finally, and most significantly, we propose that a metabolic inactivation resulting from gene erosions in *B. aphidicola*, and not by losses in the secondary endosymbiont, is behind the establishment of *S. symbiotica* as an obligate endosymbiont in the subfamily Lachninae.

## Materials and Methods

### Aphid Collection, DNA Extraction, and Sequencing

*Cinara tujafilina* aphids were collected during two consecutive years from a single *Platycladus orientalis* tuja host plant located at 39.51488 north latitude 0.42412 west longitude in the municipality of Paterna, Valencian Community in Spain. Bacteriocyte enrichment from the sample was obtained as in Gil et al. (2003) and total DNA extraction was performed following a cetyltrimethylammonium bromide method (Wilson 2002). DNA from the first year was sent for sequencing to Macrogen Inc. (Korea) where both single-end and paired-end 3-kb libraries were sequenced using 454 FLX and 454 FLX Titanium, respectively. Also an Illumina HiSeq 2000 100-bp 3-kb library was prepared with DNA from the second year and sequenced also at Macrogen Inc. (Korea).

### Preassembly

For all 454 reads, we first performed an extraction of the RAW reads using the program sff_extract v0.3.0 (http://bioinf.comav.upv.es/sff_extract/download.html, last accessed June 26, 2014), with the -l option for removing the 454 FLX titanium linker sequence, developed by the COMAV Institute (http://bioinf.comav.upv.es/index.html, last accessed June 26, 2014). Afterward, both sides of the paired-end reads were rejoined into a single read using a linker of ten undefined nucleotides (“N”). All reads shorter than 100 bp were discarded, and the remaining reads were taxonomically assigned using PhymmBL v3.2 (Brady and Salzberg 2011) with custom-added genomes of various representatives from the class Insecta (*Atta cephalotes*, *A. pisum*, *Drosophila melanogaster*, and *Tribolium castaneum*) and *Homo sapiens* GRCh37.p5, along with their corresponding mitochondrial genomes. We determined that a total of approximately 16% of the 1,033,846 reads corresponded to the *Serratia* genus (161,796 reads), as visualized using Krona v 2.2 (Ondov et al. 2011). All reads where then filtered using pyrocleaner v1.3 (Jérôme et al. 2011) with a quality threshold of 35 and minimum and maximum read lengths set to 100 and 1,000, respectively. Paired-end reads that did not match the linker sequence were used as single-end reads, the ones that matched the linker more than once were thrown away, and finally, the ones that matched only once were treated as described in (Jérôme et al. 2011).

Using FASTX-Toolkit v0.0.13.2 (http://hannonlab.cshl.edu/fastx_toolkit/, last accessed June 26, 2014), Illumina HiSeq 2000 reads were filtered for artifacts and minimum length of 50. Also right-tail clipping was performed using a minimum quality threshold of 20. Dereplication and removal of reads containing undefined nucleotides was performed using the standalone version of PRINSEQ v0.19.5 (Schmieder and Edwards 2011).

### Genome Assembly

The 454 reads were assembled using wgs-assembler v7.0 (Myers et al. 2000) with option utgGenomeSize set to 2.5 Mb and batRebuildRepeats turned on. This assembly yielded a total of 187 contigs ordered in 93 scaffolds with an N50 of 77,705 bp and a span of 2,623,798 bp (2,617,736 nongap bp). After this assembly, the scaffolds were broken and used to map reads to using MIRA v3.4.0 (Chevreux et al. 1999). This process helped to both extend contig ends and to manually inspect each one of the built contigs for inconsistencies or misassemblies resulting in 200 contigs. A custom-modified version of SSPACE v2.0 (Boetzer et al. 2011) was used to scaffold the contigs using also the HiSeq 2000 3 kb valid mate-pair data. This pipeline lead to the ordering of the aforementioned contigs into 105 scaffolds with 96 gaps.

Given the repetitive nature of this genome, as is the case for many other facultative or “recently” acquired intracellular endosymbionts, many of the gaps were flanked by repetitive regions or had them near the gaps, and manual prediction of primers avoiding repetitive sequence was time consuming. For this purpose, we developed an ad hoc program called primeScaff (http://sourceforge.net/projects/primescaff/, last accessed June 26, 2014). The program takes as input a FASTA file of the assembled scaffolds and yields GFF2 and GFF3 files with the gap and primer annotations and FASTA files of the primer sequences. This perl script iteratively uses RECON (Bao and Eddy 2002) to de novo identify repeats and mask them from the primer design step, which is accomplished using primer3 (Untergasser et al. 2012). After running this program limiting the product size to be between 100 and 1,000 bp on our scaffolds, we got primers designed for 68 out of the 96 gaps (70.83%). After inspection of the GFF3 file using UGENE (Okonechnikov et al. 2012), 39 primers were selected because they did not overlap at all any masked repetitive sequence and used for polymerase chain reaction (PCR) amplification. Out of these, 30 amplifications were positive having 24 producing reads that bridged gaps, 4 that extended contig ends but did not bridged gaps, and 2 which produced multiple amplicons probably due the fact that they fall in repetitive regions that were not identified. From the nine PCRs that failed to produce an amplicon, seven helped us identify wrongly scaffolded gaps and led to gap bridging, whereas the other two led to scaffold breaking. After this, another round of 454 read mapping on the scaffolds using MIRA v3.4.0 and visualized using Gap4 from the Staden package (Staden et al. 1999) resulted in 16 more gaps being closed. When performing the same mapping on the contigs of the scaffolds, we identified a great number of small contigs (≥200 bp and ≤2 kb) that showed clear signs of being missasemblies of repetitive-region reads, so they were removed from the database. The resulting contigs were scaffolded again, and GapFiller v1.11 (Boetzer and Pirovano 2012) was run using the mate-pair HiSeq 2000 reads resulting in 34 contigs. These contigs were scaffolded using SSPACE v2.0 into 24 scaffolds that were then submitted to primeScaff limiting the product size to be between 100 and 3,000 bp. Our script then designed six pairs of primers for the ten remaining gaps. Of these, five pairs produced amplicons, which were sequenced by Sanger and used to close five more gaps. These last 29 contigs were then used for iterative mapping of the 454 reads until no further extension was possible. Finally, after manual comparison of missing data compared with the genome of *S. symbiotica* Tucson, three more contigs were assembled and ordered into 22 scaffolds, using SSPACE v2.0, with a 454 average coverage of 13.9×.

To correct the resulting reference sequence, we iteratively ran polisher v2.0.8 (available for academic use from http://www.jgi.doe.gov/software/, last accessed June 26, 2014) on the 32 contigs using the pretreated HiSeq 2000 reads until no more corrections to the reference were made. Finally, we mapped these reads to the “polished” reference using bowtie v2.1.0 (Langmead and Salzberg 2012) and visualized the result using tablet (Milne et al. 2013) to check for signs of misassemblies or remaining sequencing errors. None were found.

### Genome Annotation and Metabolic Reconstruction

The 22 scaffolds underwent a first round of open reading frame (ORF) prediction using Prodigal v2.5 (Hyatt et al. 2010) and were annotated using BASys server v1.0 (Van Domselaar et al. 2005). tRNAs were annotated using the standalone version of tRNAscan-SE v1.3.1 (Lowe and Eddy 1997) (COVE-only) and checked using TFAM v1.4 (Tåquist et al. 2007). rRNAs were annotated using Infernal v1.1 (Nawrocki and Eddy 2013) and the Rfam database v11.0 (Burge et al. 2013) with a step of manual curation for the 16S and 23S genes using the web server of SINA (Pruesse et al. 2012) against the SILVA (Quast et al. 2013) database to correct gene boundaries. Other RNAs were also annotated using Infernal and Rfam. Insertion sequence proteins were annotated using the BLAST server from ISfinder (Siguier et al. 2006). RBSfinder (Suzek et al. 2001) was used both to correct start codons and to predict putative ribosome-binding sites of coding sequences (CDSs). Afterward, a manual curation on the annotation of genes and search for pseudogenes and other features was done on UGENE using NCBI’s BlastX, BlastP (Altschul et al. 1997), and delta-BlastP (Boratyn et al. 2012) against NCBI’s nr and nt databases as needed. Priority for these searches was as following: 1) against *Escherichia coli* K-12 substrain MG1655, 2) against *Yersinia pestis* CO92, and 3) against the whole nr database. CDSs were considered intact if no frameshifts disrupting its CDS were found and if they presented all (intact or almost intact) functional domains of the reference protein, as predicted with searches against the Pfam database v27.0 (Finn et al. 2014). If essential domains for the function were missing or incomplete, the protein was considered a pseudogene. When all features were annotated, a search of the CDSs using the standalone version of InterProScan v5.1 (Hunter et al. 2012) against the database v44.0 was done to infer Gene Ontology (GO) terms, Pfam, and InterPro motifs. Leader peptides for amino acid operons were predicted by hand and then corroborated using BLAST against their homologues in other *Serratia* genomes.

The 22 annotated scaffolds were submitted to the metabolic annotation process implemented in Pathway Tools v17.5 (Karp et al. 2010) against BioCyc and MetaCyc databases (Caspi et al. 2012). After the automatic reconstruction, manual curation of the database was done comparing to known reactions and complexes present in Biocyc.

### COG Profiles

COG functional categories were assigned using various ad hoc perl scripts to find nonoverlapping hits against the COG database using BlastP with an *e*-value cutoff of 1e-03. The COG profile displays and clustering were made using the heatmap2 function from the R package gplots. Absolute COG category frequencies were divided by the strains total number of COG-assigned CDSs. For assessing *S. symbiotica* functional divergence from the “free-living” strains, a mean of per COG category frequency was calculated for the latter and subtracted from the given category on all *Serratia* strains as in (Manzano-Marín et al. 2012).

### Single-Copy Core Genes and Phylogeny

Construction of the ortholog groups of proteins was done using OrthoMCL v2.0 (Chen et al. 2007) as in Manzano-Marín et al. (2012) using genomes available for representatives from different genera of bacteria belonging to enterobacteriaceae and Pasteurellaceae families and the current almost-finished genomes from different *Serratia* genus strains. These included *S. marcescens* strains WW4, FGI94, and Db11; *S. liquefaciens* strain ATCC 27592; *S. proteamaculans* strain 568; *S. plymuthica* strains 4Rx13 (previously classified as *S. odorifera* 4Rx13) and AS9; and *S. symbiotica* strains Tucson, SCt-VLC, and SCc. Also, representatives from other genera of Proteobacteria were used to place *S. symbiotica* SCt-VLC in a phylogeny. A complete list of organisms and their accession numbers can be found in supplementary table S2, Supplementary Material online.

The 354 single-copy proteins shared by all the strains were extracted and translated into amino acid sequences using transeq from the EMBOSS suite (Rice et al. 2000) and aligned using the L-INS-i algorithm from MAFFT v7.055b (Katoh and Standley 2013). Gblocks (Castresana 2000) was used to refine the alignment (supplementary file S2, Supplementary Material online) and a maximum-likelihood tree was calculated using 1,000 full bootstrap replicates with RAxML v7.7.6 (Stamatakis 2006) using the PROTGAMMAWAGF substitution matrix. Visual display was done using FigTree v1.4.0 (http://tree.bio.ed.ac.uk/software/figtree/, last accessed June 26, 2014) and edited in Inkscape (http://www.inkscape.org/en/, last accessed June 26, 2014).

### Genome Rearrangement and Syntheny Clusters

The aforementioned single-copy core proteins were selected to study the genome rearrangement in the *Serratia* history. For unfinished genomes, we inferred a putative scaffold/contig order aligning these versus a reference genome using MUMers (Kurtz et al. 2004) promer v3.22. Custom Perl scripts were developed to create input files for genome rearrangements plotting using genoPlotR v0.8 (Guy et al. 2010). Minimal number of rearrangements phylogenies were calculated using MGR v2.03 (Lin et al. 2010) with the circular genomes option and without using any heuristics.

Synthenic clusters were defined using the standalone version of OrthoClust (Zeng et al. 2008) taking as “seed” relationships the nonmobile single-copy core proteins shared by both *S. symbiotica* strain Tucson and SCt-VLC. From here, ad hoc perl scripts were developed to parse the outfile and yield a human-readable format to undergo manual curation of the clusters.

### Intrapopulation Rearrangements in SCt-VLC

The filtered and trimmed mate-pair HiSeq 2000 library was mapped, taking both mates separately as if single-end reads, against the final 22 scaffolds using bowtie v2.1.0. Concordant mate-pairs (RF direction and between 2,211 and 3,651 bp insert size) were discarded (∼77.69% of the paired reads). All of the cases in which only one mate or at least one partner mapped to a putative mobile region were discarded. From the 437,842 pairs mapping discordantly, approximately 92.12% mapped on the same contig. We then removed the mates that partly or entirely overlapped each other and ended up with 342,088 pairs. Then, we parsed the SAM file using an ad hoc perl script and proceeded to determine the insert size and the direction of the mates, having 1,478 in FF (∼0.43%), 3,035 in RR (∼0.89%), 11,630 in RF (∼3.41%), and 325,945 in FR (∼95.69%) orientation. Because the FF and RR orientation pairs were such few and scattered throughout the genome, we removed them because they most probably represented library errors. The FR pairs would represent typical paired-end library contamination in HiSeq 2000 mate-pair libraries. Finally, the RF (mate-pair orientation) plus FR (pair-end orientation) pairs were plotted using circos v 0.64 (Krzywinski et al. 2009).

### Genetic Degradation

First, we defined a group of CDSs that were shared by at least two *S. symbiotica* strains and complemented it with the automatic identification of shared pseudogenes using a cutoff of 30% BlastP score relative to the alignment score of the sequence with itself as described in Lerat (et al. 2003). Then, any group that had a mobile element was removed. Finally, manual curation of the groups was done, guided by gene name, genomic context, and, if necessary, manual inspection of gene-to-gene alignment. Heat map was done using the R package gplots. Histograms were also done using R.

## Results

### *The* S. symbiotica *Strain SCt-VLC Genome*

**Fig. 1.—:**
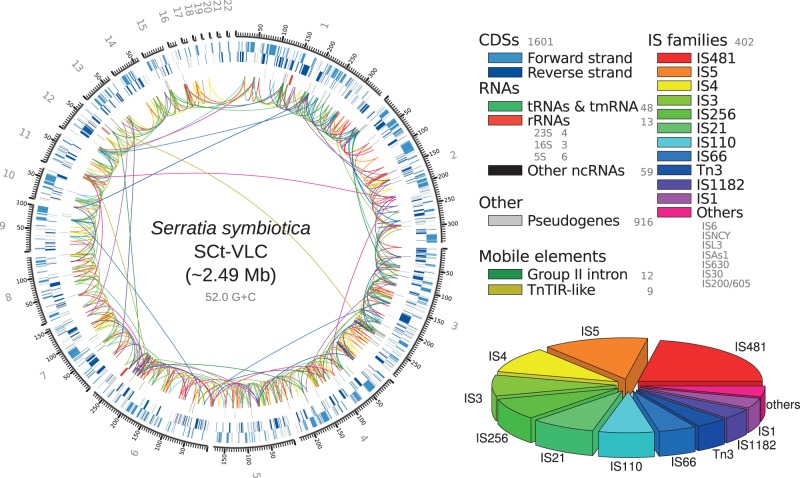
*Serratia symbiotica* strain SCt-VLC genome. *Serratia symbiotica* strain SCt-VLC genome displaying the variety and invasion by mobile element proteins. Left: Circular representation of the scaffolds composing the genome of *S. symbiotica* strain SCt-VLC. From outer to inner, the rings show features on the forward strand, features on the reverse strand, RNA features, and lines connecting different types of mobile elements. Right top: Color coding for the different elements displayed in the circular representation of the genome and their absolute abundance. Right bottom: Pie chart depicting the relative abundances of IS element proteins in the genome.

The genome of *S. symbiotica* strain SCt-VLC (hereafter SCt) (fig. 1) has been assembled to 32 contigs organized into 22 scaffolds spanning 2,494,579 bps, with an average G+C content of approximately 52% and a 454 and HiSeq 2000 average coverage of 13.90× and 632.70×, respectively. The sequences have been deposited at DDBJ/EMBL/GenBank under the accession numbers FR904230–FR904248 and HG934887–HG934889. The high level of assembly of this genome has been possible thanks to the use of various bioinformatic and experimental techniques (see Materials and Methods: Preassembly, Genome Assembly).

 Both the genome size and the G+C content are quite similar to the ones calculated for *S. symbiotica* strain Tucson (hereafter SAp), a facultative endosymbiont in *A. pisum* (table 1). In addition, SCt highly resembles SAp functionally, as determined by analysis of a COG degradation profile (supplementary fig. S1, Supplementary Material online). The genome size of SCt might differ from that of SAp given the highly fragmented genome assembly of this last genome (Burke and Moran 2011). SCt’s genome encodes for 1,601 intact putative CDSs and 916 putative pseudogenes (∼53.4% coding density), one of which is a 23S rRNA 5′ truncated gene, displaying a higher level of pseudogenization than SAp. It encodes for 1 tmRNA and 47 tRNAs, with amino acid charging potential for all 20 standard amino acids plus two tRNAs with amino acid charging potential for formylmethionine and one for lysylated isoleucine. Compared with SAp, it has lost the tRNA coding for selenocysteine. Out of the 13 rRNA genes it possesses (four, three, and six copies of the 23S, 16S, and 5S ribosomal genes, respectively), two copies of the 23S rRNA gene run-off from contig ends, being unable to determine their completeness. It presents two intact copies of the full ribosomal RNA operon, and as seen in other recently established endosymbionts (including SAp), the genome of SCt presents a high amount of mobile DNA (∼13.4% of the total genome), with a variety of IS proteins belonging to approximately 18 different families scattered throughout the genome.

**Table 1:** Comparison of Different Strains of *Serratia symbiotica* Genomes

Features	*S. marcescens* Db11	*S. symbiotica*		
		SAp	SCt	SCc
Chromosome (bp)	5,113,802	2,761,037	2,494,579	1,762,765
Mean G+C (%)	59.5	48.4	52	29.2
Predicted CDSs	4,709	2,098	1,601	672
Average CDS size (bp)	954.5	835.3	831.2	1,018.5
Pseudogenes	12	550	916	59
rRNAs (23S,16S,5S)	7,7,8	5,5,5	4,3,6	1,1,1
tRNAs	88	44	47	36
Other RNAs	69	57	59	6
CDS density (%)	87.9	56.8	53.4	38.8
Pseudogene density (%)	0.3	19.1	29.8	2.4
Mobile elements	Yes	Yes	Yes	No
Cell shape	Rod	Rod	Rod	Spherical
Lifestyle	Free living	Facultative	Co-obligate	Co-obligate

Note.—Comparison of genomic features from *S. symbiotica* strains contrasting with characteristics of a free-living relative (*S. marcescens* Db11).

**Fig. 2.—:**
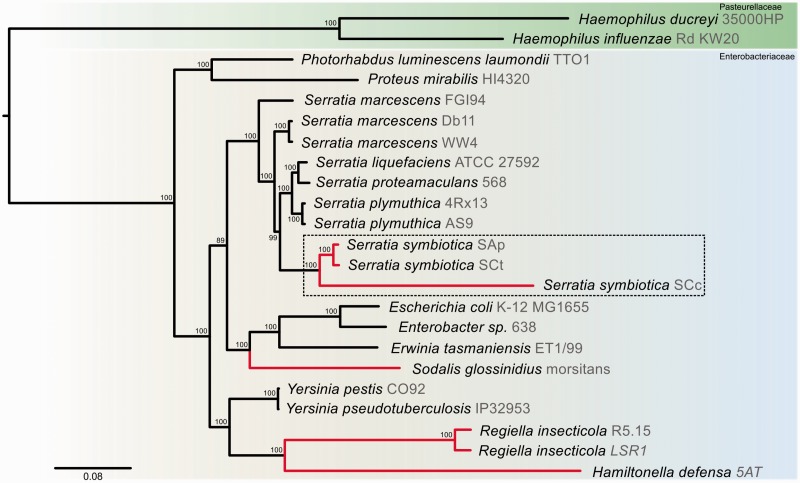
*Serratia symbiotica* strain SCt-VLC phylogenetic positioning. *Serratia symbiotica* maximum-likelihood phylogenetic reconstruction using concatenated single-copy orthologs shared by all selected strains. *Serratia symbiotica* SCt, as *S. symbiotica* SAp, has diverged less than its endosymbiotic relative in *Cinara cedri* (*S. symbiotica* SCc). The gray strain designation is showed.

We reconstructed a phylogenetic tree using 354 single-copy proteins shared among selected organisms (fig. 2) (see Materials and Methods: Single-Copy Core Genes and Phylogeny). All three *S. symbiotica* belong to the *Serratia* genus cluster and form a monophyletic group. As for SAp, the branch-length leading to SCt suggests a recent divergence from its free-living relatives. This clearly contrasts the long branch-length leading to *S. symbiotica* strain SCc (hereafter SCc), co-obligate endosymbiont with *B. aphidicola* in *C. cedri*, which shows an accelerated evolutionary process.

### Metabolic Capabilities

**Fig. 3.—:**
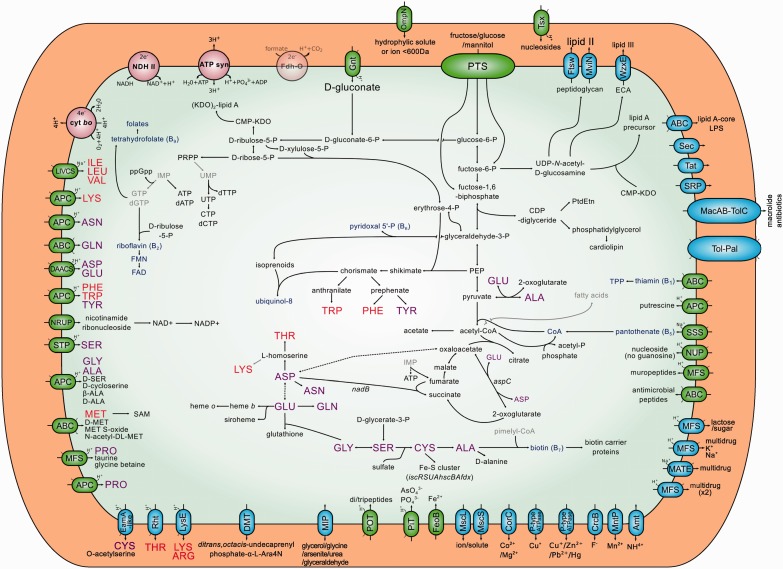
*Serratia symbiotica* strain SCt-VLC metabolic reconstruction. *Serratia symbiotica* metabolic reconstruction as done by PathwayTools. Intact pathways are shown in black lines, unclear pathways are shown in gray lines, and the ones that are already represented elsewhere with another line are shown as dotted lines. Exporters are represented with green ovals, whereas exporters and exporters/importers are represented with blue ovals. EAAs and non-EAAs are shown in red and purple lettering, respectively. Gray compounds represent those for which biosynthesis cannot be accounted for by the genomic data of *S. symbiotica* SCt-VLC. Formate dehydrogenase-O is shown semitransparent given that the pseudogenization of one of its genes is unclear, it being a selenoprotein.

Figure 3 shows the metabolic reconstruction for SCt. It is an aerobe bacterium having a complete cytochrome *bo* oxidase and, as many other endosymbionts, it can use acetyl-coenzyme A (CoA) to produce acetate and energy under oxygen-limiting conditions. It retains a complete ATP synthase, and it can grow on different carbon sources such as glucose, fructose, and mannitol, also retaining a complete phosphotransferase system (PTS) transporter for each of these sugars. Unlike SAp, it has lost the mannose PTS system and the ability to grow on trehalose and N-acetylglucosamine. It has the *treC* gene pseudogenized (responsible for the conversion of trehalose 6-phosphate to b-d-glucose 6-phosphate) in addition to a pseudogenized version of *nagA* and a deletion of the *nagB* and *nagE* genes, impairing the import and conversion of N-acetylglucosamine to fructose-6-P. As other highly reduced endosymbionts, it presents great pseudogenization in the genes involved in the tricarboxylic acid cycle (TCA) cycle. This is in clear contrast with SAp, which putatively still presents a complete cycle (Burke and Moran 2011). In this respect, it closer resembles *S. symbiotica* SCc, which, as all *B. aphidicola*, has lost almost all the genes involved in this pathway. Regarding the cell wall, *S. symbiotica* SCt retains the ability to synthesize peptidoglycan (lipid II), enterobacterial common antigen (lipid III), and lipopolysaccharides, like other facultative endosmbionts.

As for amino acid biosynthesis, it retains the capability of synthesizing four essential and nine nonEAAs. It retains complete pathways to synthesize the three aromatic amino acids, phenylalanine, tryptophan, and tyrosine, via the complete pathway starting from phosphoenol pyruvate and d-erythrose 4-phosphate. Most importantly, SCt can indeed synthesize tryptophan, not having the *trpE* and *trpG* genes pseudogenized or lost, as SAp and SCc, respectively. In addition, it is able to interconvert aspartate to glutamate (through oxaloacetate and 2-oxoglutarrate, respectively), both of which could be imported; synthesize asparagine and threonine from l-aspartate; glutamine from l-glutamate; and serine from d-glycerate-3-P, and glycine, cysteine, and alanine from l-serine. Lysine biosynthetic pathway (DAP group) presents pseudogenization in both *astC* and *argD* genes, but still retains the rest of the pathway. It could be speculated that another transaminase might be doing the job, as in the highly reduced SCc where these two genes have also been lost but the rest of the pathway (except the first and last steps) is still encoded on the genome. Similarly, in both *B. aphidicola* strains BCc and BCt, neither *argD* nor *astC* genes are present. SCt retains specific transporters for importing most amino acids it cannot synthesize, except for arginine and histidine, as is the case for both SCc and SAp strains.

With respect to cofactors and vitamins, SCt preserves complete pathways for the biosynthesis of tetrahydrofolate, flavin mononucleotide, flavin adenine dinucleotide, pyridoxal 5′P (vitamin B6), ubiquinone, and riboflavin (vitamin B2). This last vitamin is of special interest, because most *B. aphidicola* endosymbionts sequenced to date are able to synthesize it (from ribulose-5-P and GTP) (supplementary fig. S2, Supplementary Material online) and provide it to its host, which in *A. pisum* has been described as essential (Nakabachi and Ishikawa 1999). The only exceptions to this case are the *B. aphidicola* from both Lachninae subfamily representatives, *C. tujafilina* and *C. cedri*, which miss all the genes involved in this pathway, rendering *Buchnera* unable to provide riboflavin to the aphid host, and turning *Serratia* indispensable for the biosynthesis of this vitamin. Biosynthesis of other vitamins and cofactors such as thiamin pyrophosphate (TPP), biotin, and CoA could be possible given an external supply of the required intermediaries. SCt, as SCc but unlike SAp, preserves a thiamin ABC transporter (composed of the products of the *thiQ*, *thiP*, and *thiB* genes), which would in turn put a selective pressure to preserve only the genes *thiE* and *thiL* to be able to convert the imported thiamin into TPP. This is exactly the case in the more drastically reduced SCc. All this points toward the fact that it may be losing selective pressure on the thiamin biosynthetic genes, hence it is possible to speculate that the thiamin biosynthesis pathway has recently started to undergo erosion. The biotin pathway suffers from gene inactivation (missing *fabI*), although it could still synthesize biotin from 8-amino-7-oxononanoate (KAPA) by action of the *bioF* gene product, given its supplied with the precursor pimelyl-CoA or KAPA itself. Finally, regarding CoA, it could be synthesized from pantothenate, which would be imported to the cell through a pantothenate/sodium solute:sodium symporter (SSS) encoded by the *panF* gene.

Concerning the synthesis of nucleotides, a very interesting decay pattern was observed. Contrary to *S. symbiotica* SAp but similarly to SCc, the genes to synthesize inosine monophosphate from 5-phosphoribosyl 1-pyrophosphate (PRPP) have been lost or pseudogenized, whereas the genes to synthesize uridine monophosphate from PRPP have been retained. It is worth noticing that although *pyrB* is pseudogenized by a frameshift, it might still produce a functional protein. This would confer *S. symbiotica* SCt the capacity to synthesize pyrimidines de novo but not purines, which would have to be imported.

*Serratia symbiotica* SCt, as SAp, codes for a variety of translocation systems such as Sec, twin-arginine (Tat), and signal-recognition particle (SRP). It presents a complete MacAB-TolC macrolide efflux transport system, which could provide resistance via active drug efflux. It also encodes for an intact Tol-Pal cell envelope complex and a diversity of export systems for a variety of compounds. This richer repertoire of translocators is in clear contrast with SCc, which only encodes for the Sec and SRP systems.

### Genome Rearrangements and Mobile Elements

**Fig. 4.—:**
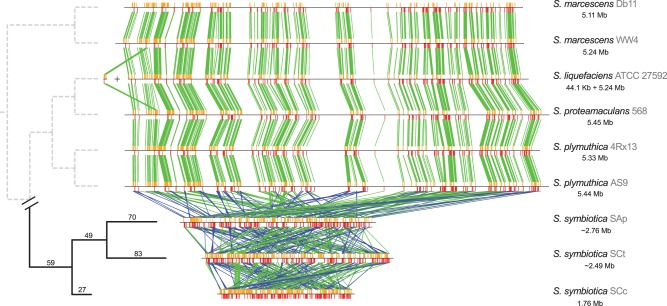
*Serratia symbiotica* minimum number of rearrangements tree of the single-copy core genes of the genus *Serratia.* Left: Rooted minimum number of rearrangements tree as calculated by MGR. Right: Pairwise synteny plots of free-living *Serratia* along with endosymbiotic relatives *S. symbiotica* strains SAp (*A. pisum*), SCt (*C. tujafilina*), and SCc (*C. cedri*). Species names (the strain designation is showed in gray) are shown along with its genomic size in Mb.

Given the high presence of mobile genetic elements in *S. symbiotica* SCt, we explored the history of the rearrangements suffered in the branch leading to the *S. symbiotica* clade. As reported before (Burke and Moran 2011; Lamelas et al. 2011; Manzano-Marín et al. 2012), through a minimal rearrangement phylogeny we established the distances among the *S. symbiotica* strains (fig. 4). As expected, although no rearrangements are observed among the free-living *Serratia*, a great number of them has led to the *S. symbiotica* clade. Unexpectedly, we found a great number of rearrangements suffered even between *S. symbiotica* SCt and SAp. Furthermore, a similar number of rearrangements was found when scaffolds belonging to SAp were arranged taking SCt’s scaffolds as reference (supplementary fig. S3, Supplementary Material online), thus discarding a bias due to the free-living *Serratia* genome selected for arranging SCt and SAp’s scaffolds. This clearly contrasts with the fact that phylogenetically these two strains find themselves extremely close together, not having diverged greatly since the last common ancestor.

Among the different mobile genetic elements SCt possesses (fig. 1), IS481 is the most abundant. Nevertheless, we were also able to find many other types that are also shared by SAp. Among these, a similar newly characterized mobile quorum-sensing system termed TnTIR in *S. marcescens* strain SS-1 (Wei et al. 2006a, 2006b) was identified. This mobile element is also present but not characterized in SAp as well as a group II intron mobile element (GIIME) (retrotransposon). This latter is also found in the genomes of other two facultative endosymbionts of aphids (*R. insecticola* strain LSR1 and *H. defensa* strain 5AT).

To explore how the high presence of mobile genetic elements might have impacted genome rearrangement in *S. symbiotica*, we compared SCt and SAp’s genomes. SCc’s genome was not taken into account because it diverges significantly from the other two strains and lacks any traces of mobile elements. We defined a set of 165 syntenic clusters shared by both *S. symbiotica* strains, with an average size of 9,596.90 bp (for strain SCt). In addition, we determined which types of elements are present in the close vicinity of these syntenic clusters (±3 kb). The great majority (129) are flanked on at least 1 side by an IS gene, 22 by putative phage elements, 11 by GIIMEs, and 4 by TnTIR-like elements (supplementary fig. S4: inner rings, Supplementary Material online). In some instances (when information available from SAp), both *S. symbiotica* SCt and SAp strains displayed the same mobile elements flanking the syntenic clusters. This would imply that the same mobile elements have mediated different rearrangements in these two organisms.

To inquire into intrapopulation rearrangements, we mapped all HiSeq 2000 nonrepetitive paired-reads and extracted the ones that mapped discordantly (supplementary fig. S4: outer rings, Supplementary Material online) (see Materials and Methods: Intrapopulation rearrangements in SCt). Basically, these reads were composed of paired-end library contamination and, we were unable to find any major indication of intrapopulation rearrangements, as determined by the lack of important clusters of paired reads indicating different genome organizations.

**Fig. 5.—:**
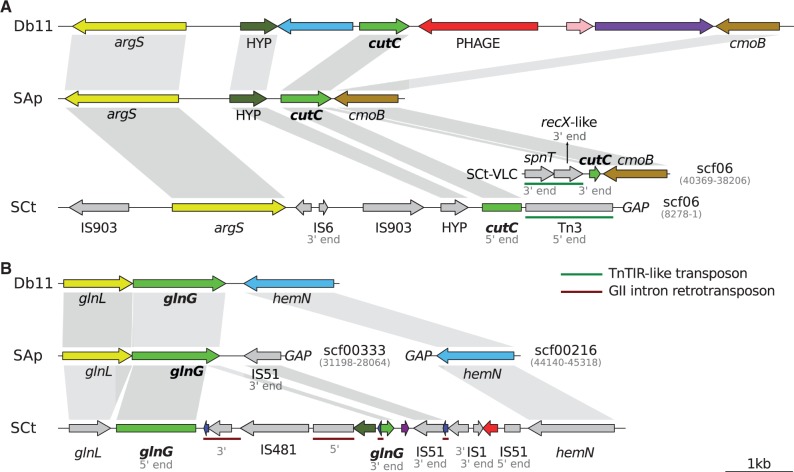
Mobile-genetic element driven genetic inactivation. Examples of genes are shown where one can evidence the inactivation and genome rearrangement driven by mobile genetic elements. (*A*) Translocated *cutC* gene; (*B*) interrupted *glnG* gene. Comparison is shown from the free-living *S. marcescens* Db11 to the facultative *S. symbiotica* SAp to the co-obligate *S. symbiotica* SCt. Color arrows represent intact CDS and gray ones pseudogenes. Genes of interest are always colored.

In addition to frameshifts, mobile elements have been proposed as a minor driving force of gene inactivation and genome size reduction in young endosymbiont genomes (Belda et al. 2010; Oakeson et al. 2014). We have found at least 11 genes that are split by mobile elements into different parts across the genome. Even in some cases, whereas *S. symbiotica* SAp presents an intact CDS or pseudogene, SCt presents an interrupted (fig. 5*A*) or translocated (fig. 5*B*) version caused by one or more mobile elements. Additionally, seven cases of this type of inactivation were found, but given the nature of the inactivated protein or the lack of comparable sequence in SAp, we were unable to determine if these inactivations were unique to SCt strain. Additionally, many pseudogenized mobile-element proteins form “genomic wastelands” composed of a variety of inactivated proteins ordered in tandem.

### Decay of Amino Acid Biosynthesis Operons and Genes

**Fig. 6.—:**
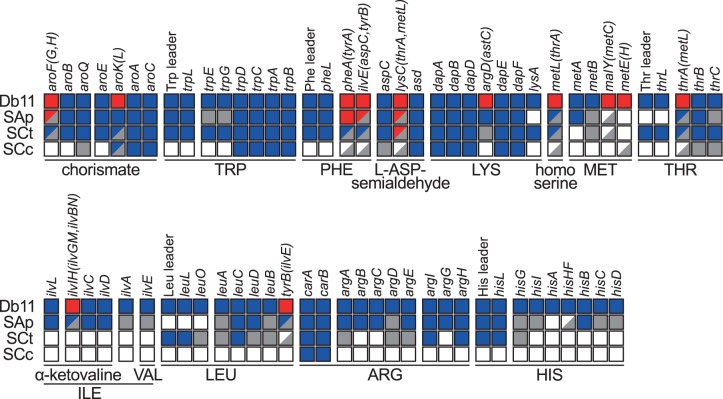
Erosion of amino acid biosynthetic genes in *Serratia symbiotica* endosymbionts. Inactivation tables for erosion in amino acid biosynthesis pathways in *S. symbiotica* endosymbionts compared with the free-living *S. marcescens* Db11. A clear graduation in degradation and redundancy is evidenced from *S. marcescens* Db11 to *S. symbiotica* SCc. Boxes represent genes. In red color meaning redundancy, blue meaning one active gene, gray a pseudogenized gene, and white an absent gene. Half-colored boxes in redundant functions mean different states in each one of the redundant units.

Because the main role of *B. aphidicola* as an obligate endosymbiont is to supply EAA lacking from the aphid diet, the degradation of these routes in the other endosymbionts coexisting with *B. aphidicola* could give us insights into their level of accommodation with the already present *Buchnera* endosymbiont. This is evidenced by the difference in degradation of the genes involved in these metabolic functions between the facultative SAp and the obligate SCc (Burke and Moran 2011; Lamelas et al. 2011). We manually reannotated the genes involved in the synthesis of EAA in all three *S. symibotica* genomes as well as their leader peptides and leader sequence elements (which function as regulatory elements attenuating the expression of the genes in the operon in response to levels of expression of it [Kolter and Yanofsky 1982]) using a combination of BlastX (against NCBI’s nr database) and cmsearch from the infernal package (Nawrocki and Eddy 2013) (against the Rfam database [Burge et al. 2013]) (fig. 6).

We found a gradual degradation (from free-living *S. marcescens* Db11 to *S. symbiotica* SCc) of some of the genes involved in the biosynthetic pathways of arginine, isoleucine, and valine, all of which can be synthesized by *B. aphidicola* in cooperation with the aphid host (Hansen and Moran 2011; Lamelas, Gosalbes et al. 2011; Lamelas et al. 2011). Additionally, we found some operons and units whose structures are disrupted or shortened in the *S. symbiotica* endosymbionts, sometimes eliminating their regulation by the absence of leader attenuators, peptides, and/or regulatory proteins. An example of these is the *thrABC* (threonine) biosynthesis operon. In *S. marcescens* Db11, the *thrABC* operon presents upstream its respective leader sequence and peptide (*thrL*). However, in Sap, both the leader sequence and peptide seem to have been eroded, while in SCt, the *thrABC* genes have been physically separated from its leader sequence attenuator and peptide into different locations in the genome by the action of an IS1182 family protein (supplementary fig. S5*A*, Supplementary Material online). Another example lies with the contiguous *argE* and *argCBGH* (arginine) biosynthetic gene units. While intact in *S. marcescens* Db11 and in SAp, SCt has an extremely shortened version of the second unit, retaining only the *argE* and *argH* genes with a mere 179 bp between them (supplementary fig. S5*B*, Supplementary Material online). Additional examples are the *Ilv* (isoleucine) (supplementary fig. S5*C*, Supplementary Material online), *hisGDCBHAFI* (histidine) (supplementary fig. S5*D*, Supplementary Material online), and *leuABCD* (leucine) biosynthetic operons (supplementary fig. S5*E*, Supplementary Material online) which also present disrupted and shortened structures, missing or having pseudogenized versions of some genes in the SAp and SCt endosymbionts. All of these features point toward a greater accommodation of *S. symbiotica* SCt to its *Buchnera* partner when compared with SAp.

### *Genetic Erosion in Different* S. symbiotica *Strains*

To gain some insight into the functional categories that are being affected by the different genetic degradations suffered by the *S. symbiotica* strains, we clustered and manually curated a list of all shared nonmobile genes that appeared in at least two of the three endosymbiotic strains. We then identified the state of each one of these genes and performed a two-way clustering to identify different sets of genes (supplementary fig. S6, Supplementary Material online). Regarding the sets including a pseudogenized or absent gene in SCc, we find three main groups where we are able to infer in which strain, the pseudogenization event happened. These sets comprised an intact CDS in SCt or SAp and a pseudogene in the remainder, and an intact CDS in both SCt and SAp. We noticed there is a very similar distribution of the genes in these three different categories, having the ones involved in metabolism and cellular processes and signaling being the most affected ones followed by the poorly characterized genes. This trend comes as expected for this sets because both SCt and SAp strains still retain a more complex metabolism and a set of proteins involved in cell architecture. Meanwhile, the highly reduced coprimary SCc strain has lost many genes in these categories given its obligate intracellular lifestyle, probably due to the deep accommodation into the *Buchnera*-aphid symbiotic system. Finally, it is also worth emphasizing that the set comprised an active CDS in SAp, a pseudogene in SCt and a missing gene in SCc, is larger than that where the pseudogene is held by SAp and the intact CDS by SCt, meaning a great number of genes being lost in SCt’s branch, although again, the pseudogene definition in both annotations can be a factor influencing this result.

## Discussion

Secondary endosymbionts present diverse characteristics differentiating them from their free-living relatives. In insects, they generally show a great enrichment in mobile elements, a lower G+C content and an intermediate genome size between that of their free-living kins and typical obligate endosymbionts (Degnan et al. 2009; Newton and Bordenstein 2011; Oakeson et al. 2014). From the different secondary endosymbionts aphids can harbor, *S. symbiotica* presents a very particular and intriguing case, because it has been found so far in different stages in distinct aphids (Burke and Moran 2011; Lamelas et al. 2011). In the pea aphid *A. pisum*, *S. symbiotica* is facultative, extracellular, and with a genome rich in mobile elements that is about half the size of that of a free-living *Serratia*. In the cedar aphid *C. cedri*, a deep association has been formed between *S. symbiotica* and *B. aphidicola*, with the former now being a co-obligate intracellular endosymbiont with a highly reduced genome size of only about 30% of that of a free-living *Serratia*, deprived of mobile elements and with more than half of its genome showing no traces of any functional sequence. In previous works comparing free-living *Serratia* against a facultative *S. symbiotica* (SAp) and a co-obligate one (SCc) (Lamelas et al. 2011; Manzano-Marín et al. 2012), we determined that *S. symbiotica* SCc was a missing link between a facultative and an obligate endosymbiont. Here, we have shown that *S. symbiotica* SCt from *C. tujafilina* is yet another intermediate stage in the process of accommodation into the previously existent *B. aphidicola*-aphid consortium. Despite both SCt and SAp being morphologically rod-shaped and located in sheath cells, secondary bacteriocytes, and extracellularly (Fukatsu et al. 2000; Moran, Russell, et al. 2005; Lamelas et al. 2008) (supplementary fig. S7, Supplementary Material online). Genomically, SCt evidences greater gene inactivation, having a lower number of predicted intact CDSs and a higher one of pseudogenes, although this might be partly influenced by the standards in genome annotation. Also, when comparing gene degradation, we were able to determine that most of the missing or pseudogenized genes in the highly eroded *S. symbiotica* SCc (Lamelas et al. 2011), appear to be active in one or both SCt and/or SAp strains (supplementary fig. S6, Supplementary Material online). These mainly belong to the functional processes of metabolism, cellular process, and signaling as well as to the poorly characterized set of genes. These functional categories are highly reduced in many tiny genomes from obligate intracellular bacteria such as *S. symbiotica* SCc or *B. aphidicola* (McCutcheon and Moran 2012). We also observed, though in a lower abundance, the degradation of genes in information storage and processing, indicating a reduction in the cell core machinery.

The role mobile elements play in the genome reduction process suffered by bacteria undergoing a change of lifestyle from free-living to obligate mobile-element-deprived endosymbionts, has been typically derived from performing comparative studies between ancient endosymbionts showing no mobile elements and related free-living bacteria, with a “controlled” number of these. Lately, some examples of recently acquired endosymbionts being compared with their free-living relatives have been published (Burke and Moran 2011; Oakeson et al. 2014). However, most interesting is the comparison of two phylogenetically very closely related strains enriched in mobile elements, as is the case of *S. symbiotica* SAp and SCt. By studying the genome rearrangements and the impact that mobile elements have had on the genome architecture of SCt, we can confidently determine that those belonging to IS families have been the key factor promoting massive rearrangements. These mobile genetic elements have also mediated inactivation in various genes, sometimes creating long stretches of inactivated proteins in tandem. In addition, the study of intrapopulation rearrangements indicated that in a certain population of *C. tujafilina* aphids, the genome of SCt is quite stable in a particular point in time, despite the great availability of mobile substrate. This would mean rearrangements might be happening in a slow fashion, although periodic and/or single cell resequencing might be needed to determine the fixation rates and variations of these in a population. The great number of rearrangements separating each *S. symbiotica* in a minimal rearrangement phylogeny (fig. 4) let us postulate that we are seeing three divergent lineages of *S. symbiotica*. These would each suffer a particular genome reduction process that would finish giving different architectures for each genome, while maintaining microsynteny. Although at random at the beginning of the mobilization process, purifying selection will occur at a certain point to avoid the loss of essential genes.

Metabolically, *S. symbiotica* SCt, just as both SAp and SCc strains, seems to be mainly in charge of producing vitamins and cofactors. SCt also presents a variety of pathways that have been inactivated or lost compared with SAp. Such pathways mainly include the biosynthesis of thiamin (for which it retains an ABC transporter), the loss of the mannose PTS system, and the ability to grow on trehalose and n-acetylglucosamine. Most importantly, *S. symbiotica* SCt presents a degraded TCA cycle, which clearly contrasts with SAp but resembles SCc (Lamelas et al. 2011) and other ancient genomically reduced endosymbionts such as *B. aphidicola* (Lamelas, Gosalbes et al. 2011). In general, *S. symbiotica* SCt reflects a very similar but slightly more reduced metabolic set of functions when compared with SAp. Nevertheless, as indicated by the phylogenomic reconstruction, these two genomes are very closely related, thus the key of the differences in the gene loss state must be related to the different environment where they live and/or the roles their *B. aphidicola* partners play in the nutrient provisioning. Addressing this fundamental question, we have been able to identify the production of riboflavin (vitamin B2) as that which would hold the key to the persistent presence of a second obligate endosymbiont in the Lachninae subfamily, *S. symbiotica* (Lamelas et al. 2008). Although in *A. pisum*’s *Buchnera* (presenting less genome reduction) the genes involved in the pathway for the biosynthesis of riboflavin are all present, the complete inactivation of this pathway has only been found so far in the functionally similar (supplementary fig. S8, Supplementary Material online) and reduced *Buchnera* endosymbionts from aphids belonging to the Lachninae subfamily (*C. cedri* and *C. tujafilina*). Both of these present an association with a *S. symbiotica* endosymbiont, which is able to synthesize this compound, and thus making its presence obligatory. Given the phylogenetic evidence pointing toward an obligate status for *S. symbiotica* in many members from the Lachninae subfamily (Lamelas et al. 2008), we can speculate that this loss happened in the *Buchnera* genome from the common ancestor of the Lachninae. This loss could have been propelled by the constant association with a *Serratia* facultative endosymbiont, holding an intact set of genes for this route. *Serratia symbiotica* would then have become mandatorily present, further driving gene losses in both *Buchnera* and *S. symbiotica* partners. In the particular case of *C. cedri*, *B. aphidicola* would then have experienced even greater losses deriving from this constant association, mainly the one rendering it unable to supply the EAA tryptophan (Gosalbes et al. 2008). This could have reinforced the establishment of *S. symbiotica* as a co-obligate intracellular endosymbiont while at the same time driven further its genome degradation. On the contrary, neither in the *B. aphidicola* from *A. pisum* nor in the one from *C. tujafilina* this is observed and both *Buchnera* preserve all genes necessary to synthesize tryptophan. It is worth noticing that BCt is the only *Buchnera* so far reported to have a chimeric Leu/Trp plasmid (Gil et al. 2006) without the typical *trpEG* gene expansion, postulated to serve for amplification of tryptophan synthesis (Lai et al. 1994). Along with this fact, SCt is the only *S. symbiotica* sequenced so far that presents intact copies of the *trpEG* genes. This displays an evident coevolution of these two endosymbionts in their respective aphid host.

In summary, we have found evidence that let us conclude that the genome of *S. symbiotica* from *C. tujafilina* finds itself in a stage of accommodation intermediate between that of facultative SAp and obligate SCc. This represents an especially interesting case in which two genomically very similar bacteria have different dispensability status dictated mainly by their obligate partner, *B. aphidicola*. *S. symbiotica* SCt-VLC could represent the very first stages of the settling down process from a facultative to a reduced obligate intracellular endosymbiont, not having yet experienced the massive losses leading to a deeply rooted co-obligate endosymbiosis as witnessed in the symbiotic system of *C. cedri*.

## Supplementary Material

Supplementary files S1 (tables S1 and S2), S2, S3 (figs. S1–S8), and S4 are available at *Genome Biology and Evolution* online (http://www.gbe.oxfordjournals.org/).

Supplementary Data
