# Supplementary material for: Settling Down: The Genome of Serratia symbiotica from the Aphid Cinara tujafilina Zooms in on the Process of Accommodation to a Cooperative Intracellular Life
Source: Genome Biol Evol. 2014 Jun 19;6(7):1683–98. doi: 10.1093/gbe/evu133 (PMC4122931; doi:10.1093/gbe/evu133)
Supplement: Supplementary Data [file supp_6_7_1683__index.html]

Settling down: The genome of Serratia symbiotica from the aphid Cinara tujafilina zooms in on the process of accommodation to a cooperative intracellular life — Settling Down: The Genome of Serratia symbiotica from the Aphid Cinara tujafilina Zooms in on the Process of Accommodation to a Cooperative Intracellular Life — Supplementary Data 

# Settling Down: The Genome of *Serratia symbiotica* from the Aphid *Cinara tujafilina* Zooms in on the Process of Accommodation to a Cooperative Intracellular Life

## Supplementary Data

files

**Files in this Data Supplement:**

- Supplementary Data - zip file
