# Supplementary material for: Settling Down: The Genome of Serratia symbiotica from the Aphid Cinara tujafilina Zooms in on the Process of Accommodation to a Cooperative Intracellular Life
Source: Genome Biol Evol. 2014 Jun 19;6(7):1683–98. doi: 10.1093/gbe/evu133 (PMC4122931; doi:10.1093/gbe/evu133)
Supplement: Supplementary Data [file supp_evu133_suppl_data.zip › suppl_file_3.pdf]

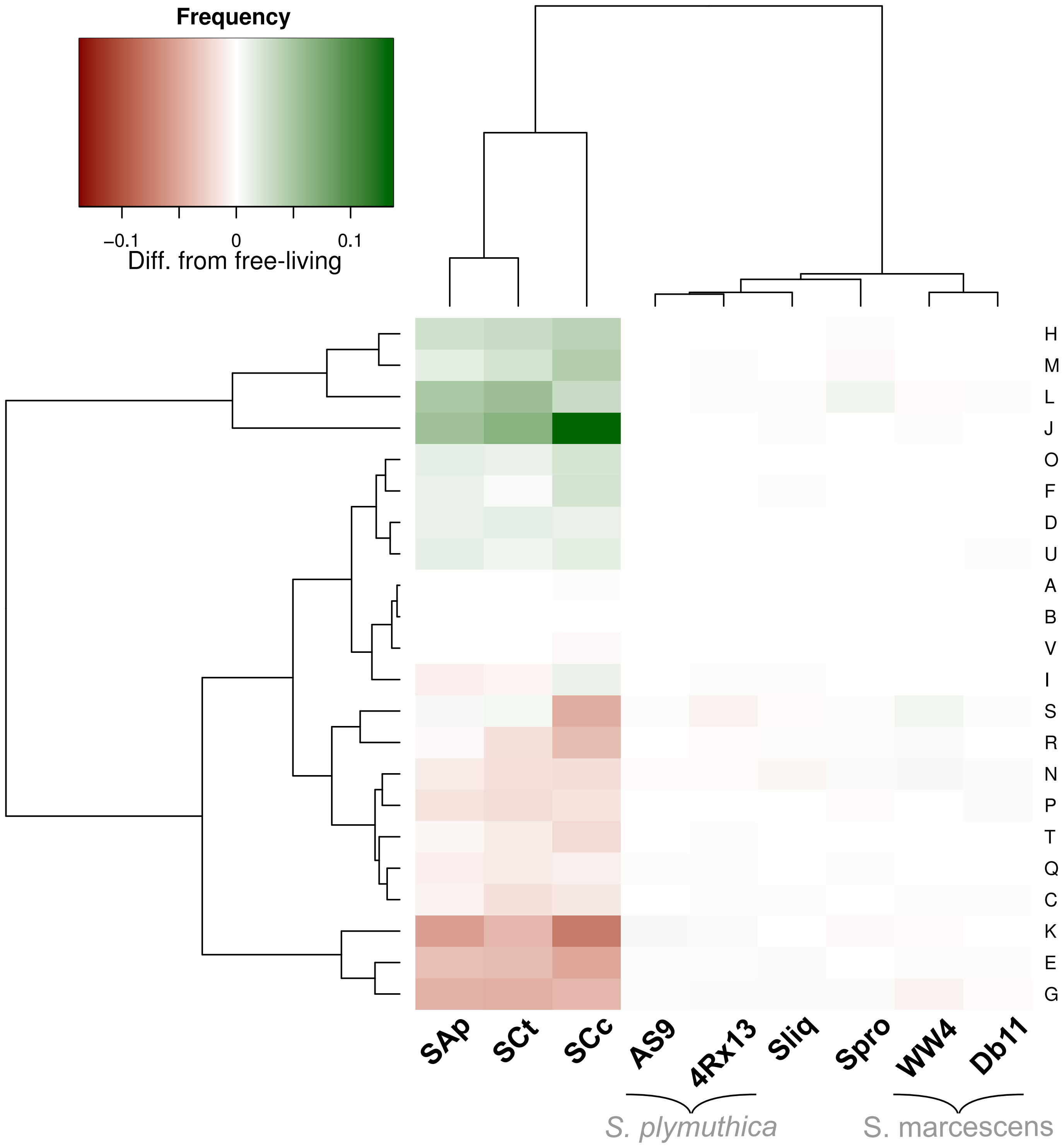


**Fig. S1. Functional profile disruption in *S. symbiotica***
Heat map showing the two-way clustering of the COG profiles frequency divergence from the free-living *Serratia* averages. On the right side of the heat map, one-letter COG assignments for each row are displayed. On the bottom of each column, abbreviations for each of the *Serratia* strains are as in supplementary **table S2**.

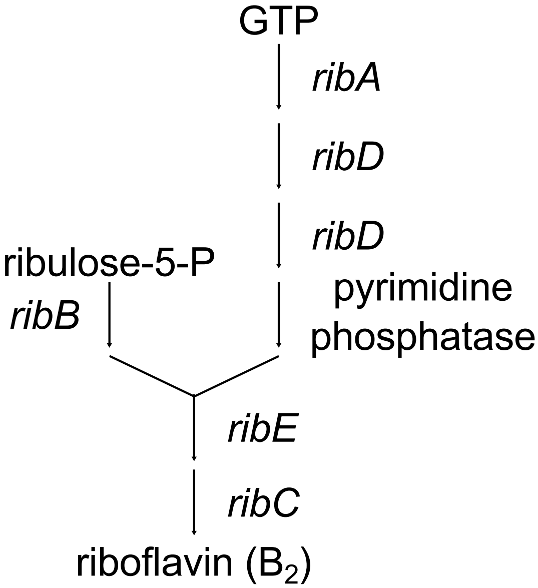


**Fig. S2. Riboflavin biosynthetic pathway**

Riboflavin biosynthetic pathway from ribulose 5-P and GTP, displaying the genes involved in each reaction on the right using italic letters. Gene for pyrimidine phosphatase is assumed to exist but has not been described. Each arrow corresponds to one reaction.

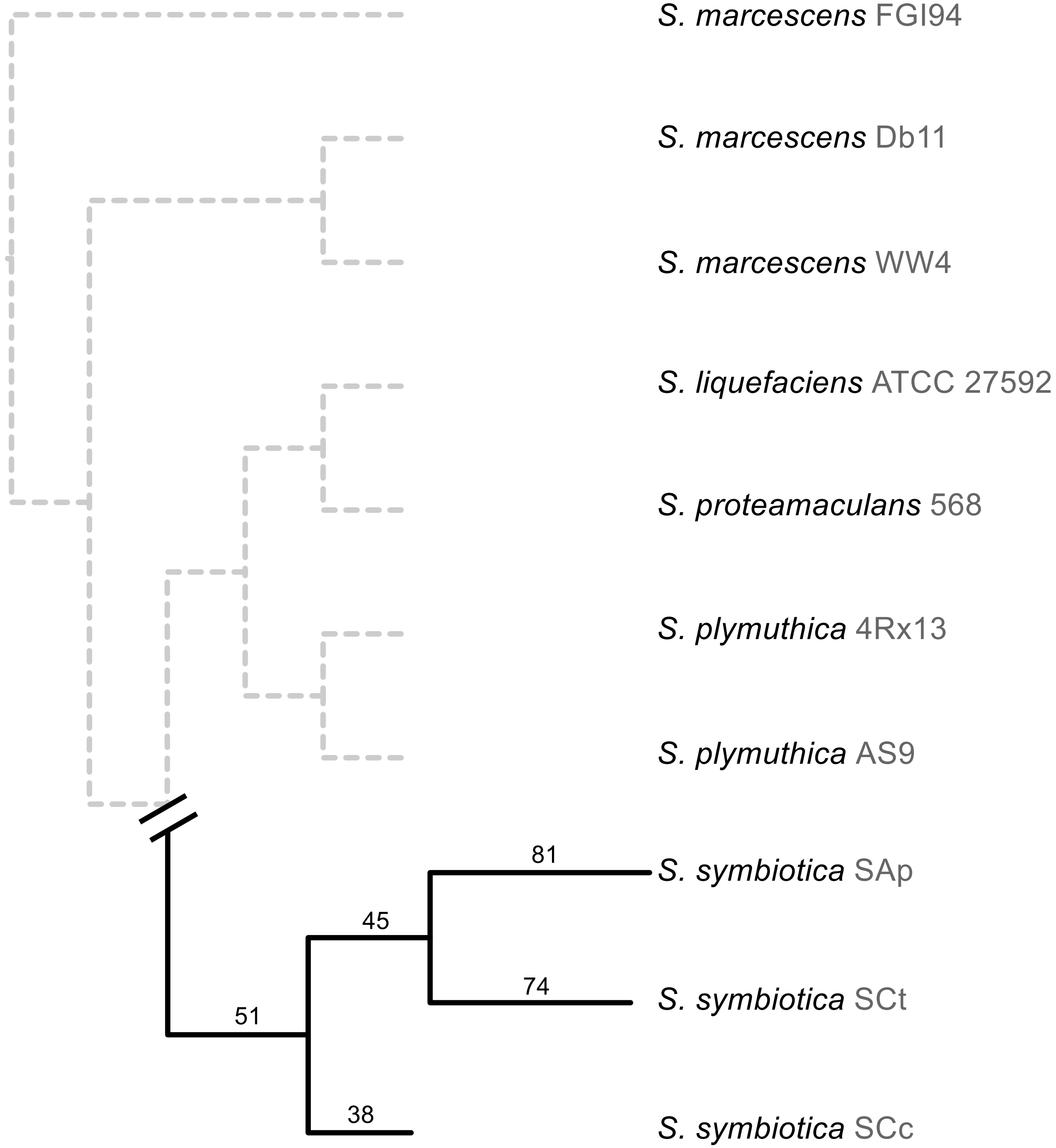


**Fig. S3. *S. symbiotica* minimum number of rearrangements tree of the single-copy core genes of the genus *Serratia* based on SCt-VLC genome order**

Rooted minimum number of rearrangements tree as calculated by MGR. Scaffold order for *S. symbiotica* SAp determined using SCt as reference.

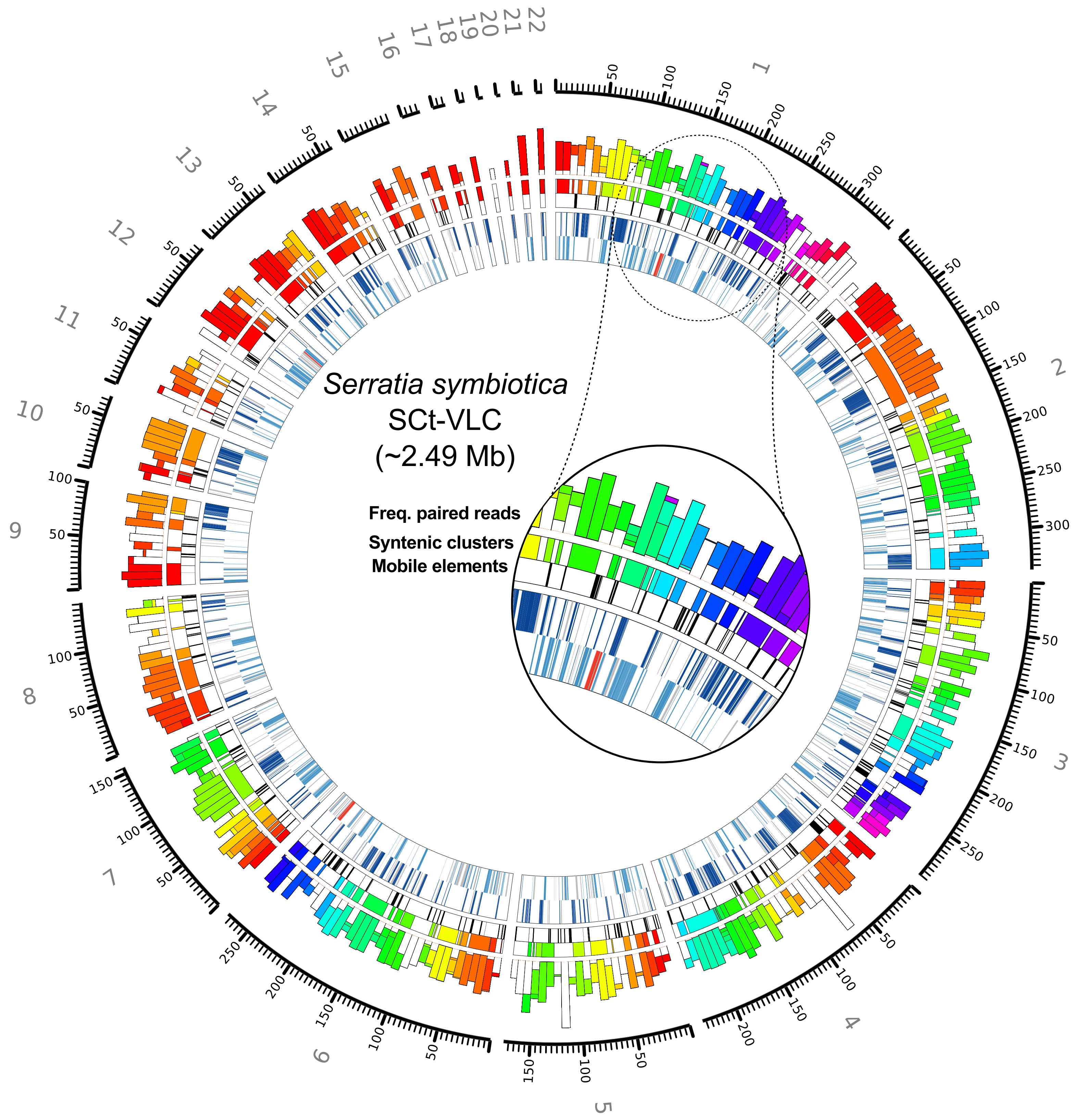


**Fig. S4. S. symbiotica discordant paired-end reads and syntenic clusters**

Discordant paired-end alignments for *S. symbiotica* SCt-VLC scaffolds displaying no detectable intra-population rearrangements. The outer ring represents the absolute abundance of paired-end reads color-coded according to the synteny cluster on the same scaffold their mate occurs at. From outer to inner, the rest of the rings represent the color-coded synteny clusters, the mobile-element proteins in black, the forward strand features and the reverse strand features (color-coded as in **fig. 1**), respectively.

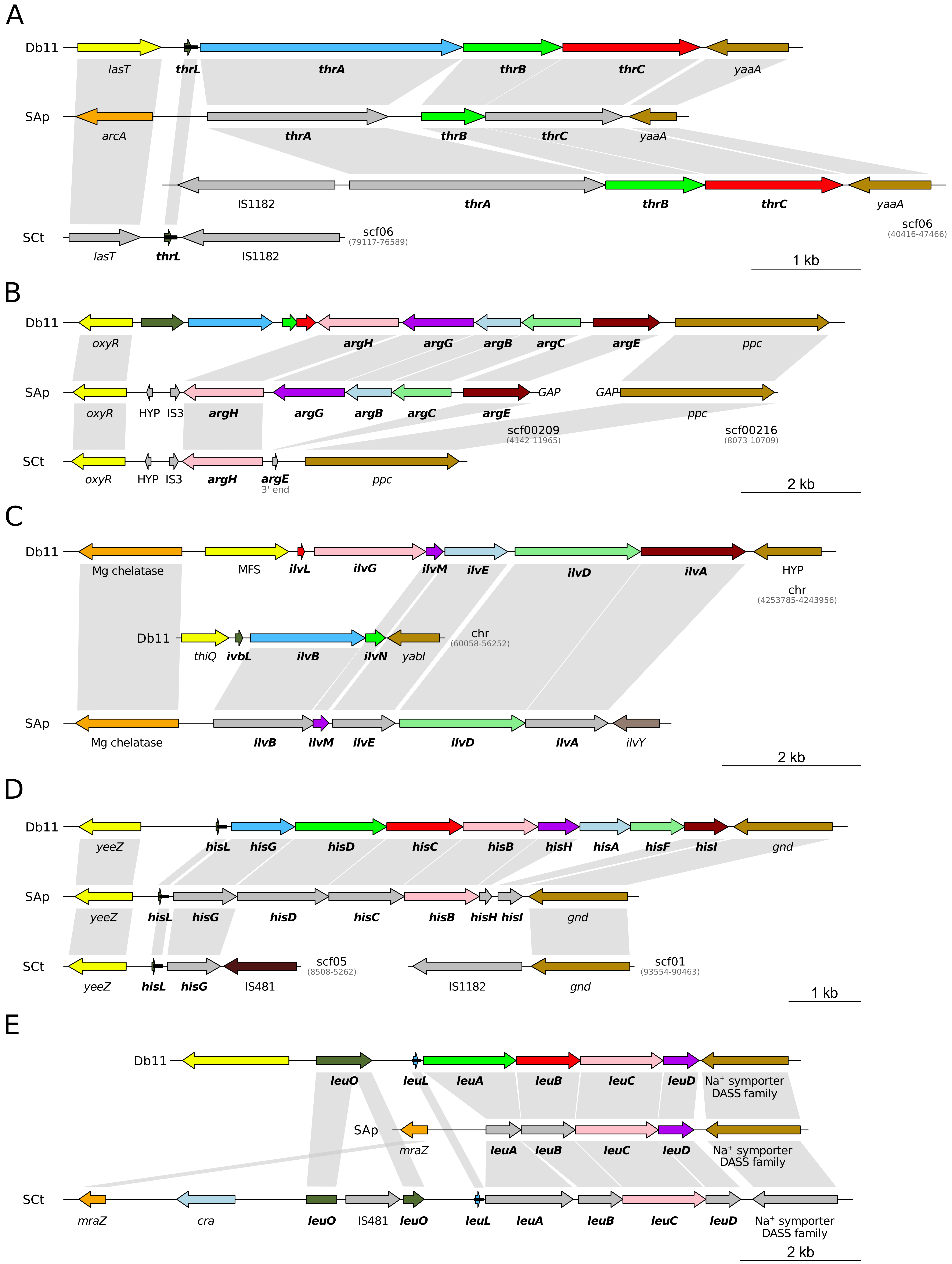


**Fig. S5. *Serratia* byosinthetic operons and transcription units degradation in *S. symbiotica***
Diagrams displaying the erosion of biosynthetic operons and transcription units in the *S. symbiotica* SCt and SAp strains. **A:** *thrABC* operon; **B:** *argHGBC* operon and *argE* transcription unit; **C:** *ilvBN* and *ilvGmDA* operons; **D:** *hisGDCBHAFI* operon; **E:** *leuABCD* operon. Color arrows represent intact CDS, grey ones pseudogenes and black bars attenuator leader sequences.

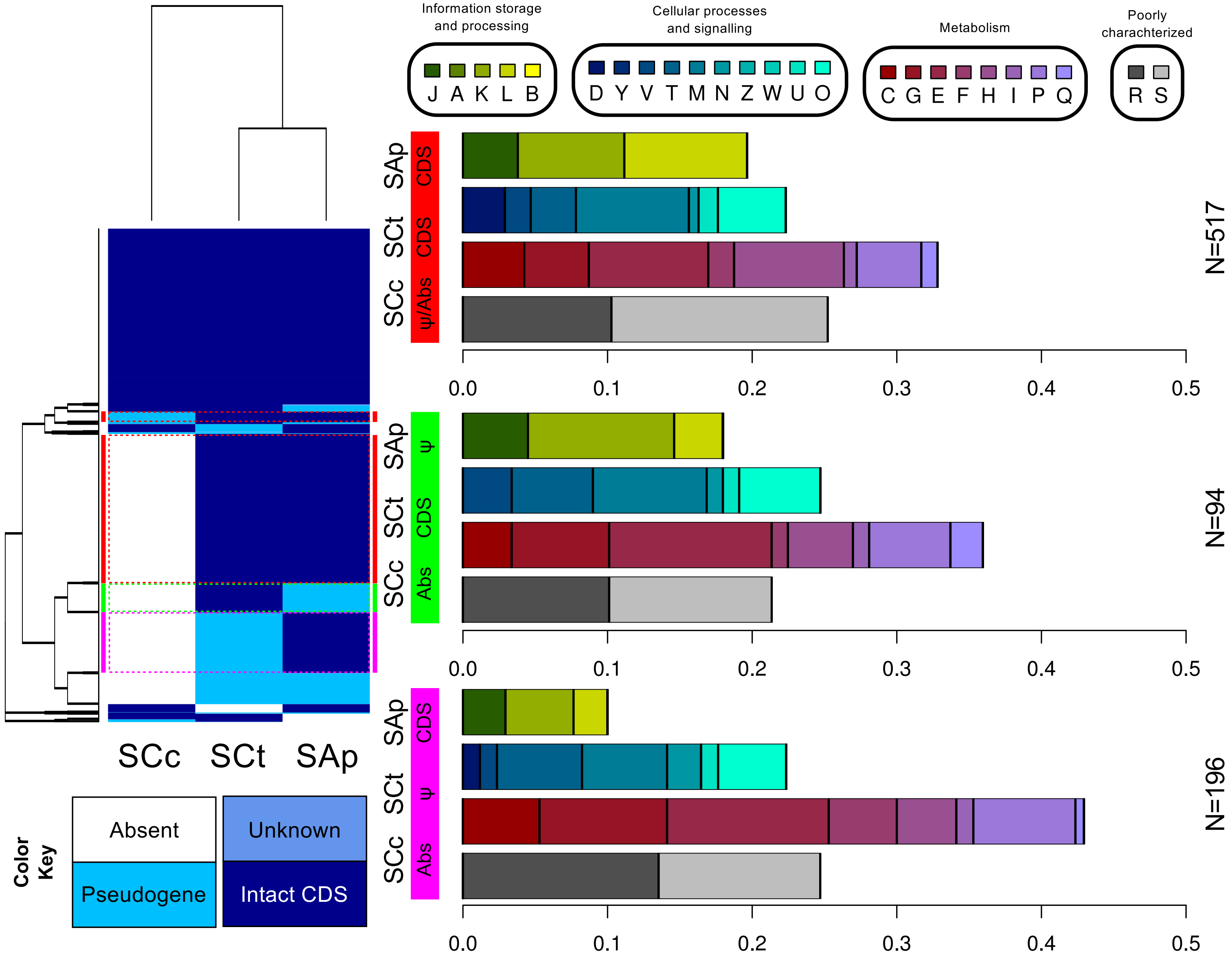


**Fig. S6. Genetic erosion among S. symbiotica endosymbionts** Different genetic inactivations suffered by the three *S. symbotica* endosymbionts: SCc (*C. cedri*), SCt (*C. tujafilina*) and SAp (*A. pisum*). **Left:** Heat-map of the number-coded state of genes shared by at least two *S. symbiotica* strains. In dotted squares are the regions represented in the histograms. **Right:** histograms of each one of the highlighted regions of the heat-map, indicating the state of the genes in each strain (Abs for absent, ψ for pseudogene and CDS for intact protein-coding gene). Each horizontal bar represents a broad COG category and is color-coded by the one-letter COG categories as explained in the top right. On the far right the number of genes representing each regions' histogram is given. Abbreviations for each of the *S. symbiotica* strains are as in supplementary **table S2**.

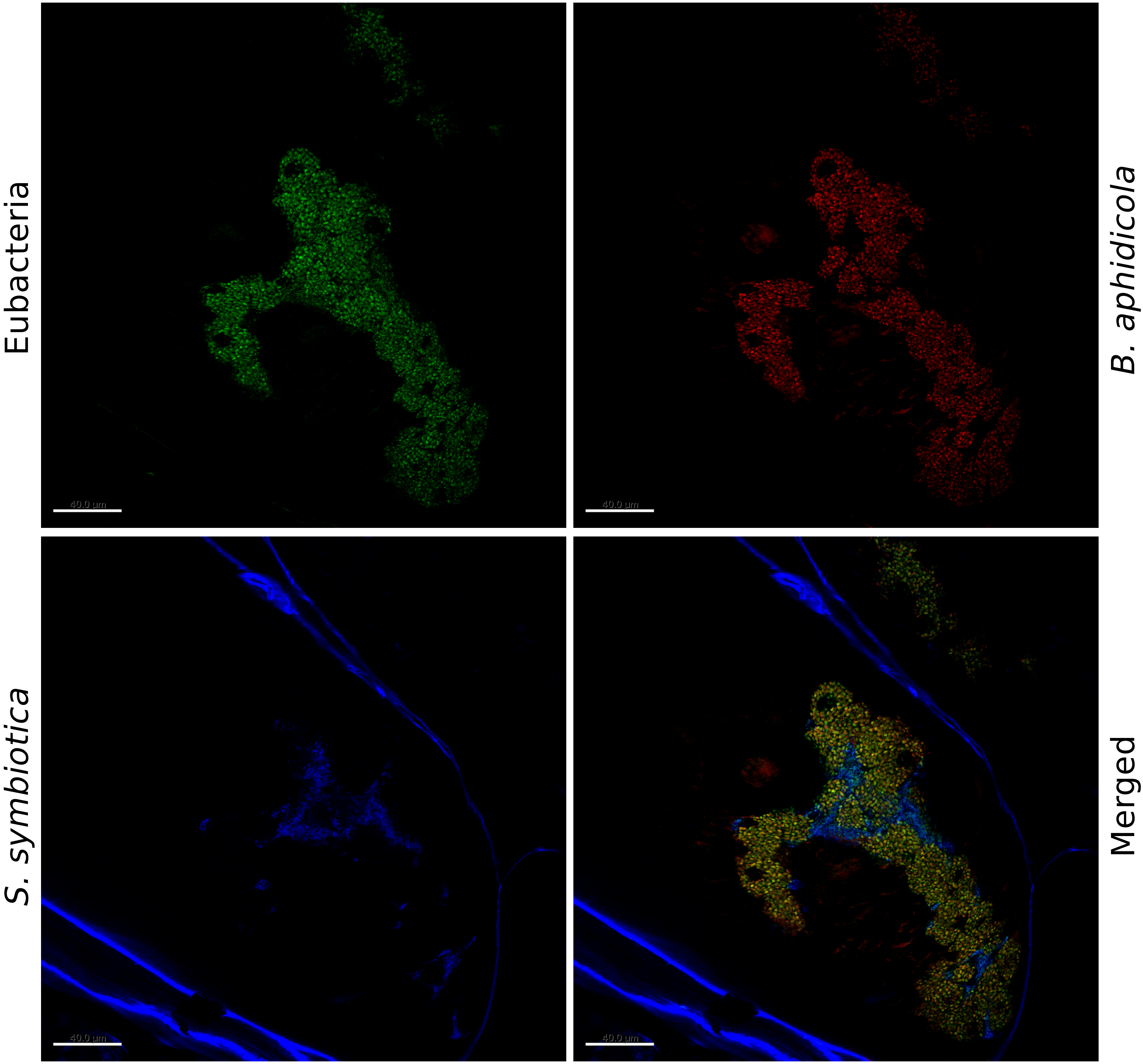


**Fig. S7. Location of both *B. aphidicola* and *S. symbiotica* endosymbionts in embryos of the aphid *C. tujafilina***

FISH image of an embryo of *C. tujafilina* displaying the location of *B. aphidicola* confined to bacteriocytes (red) and *S. symbiotica* in sheath cells and bacteriocytes (blue). White bar at the bottom left corresponds to 40.0 µm

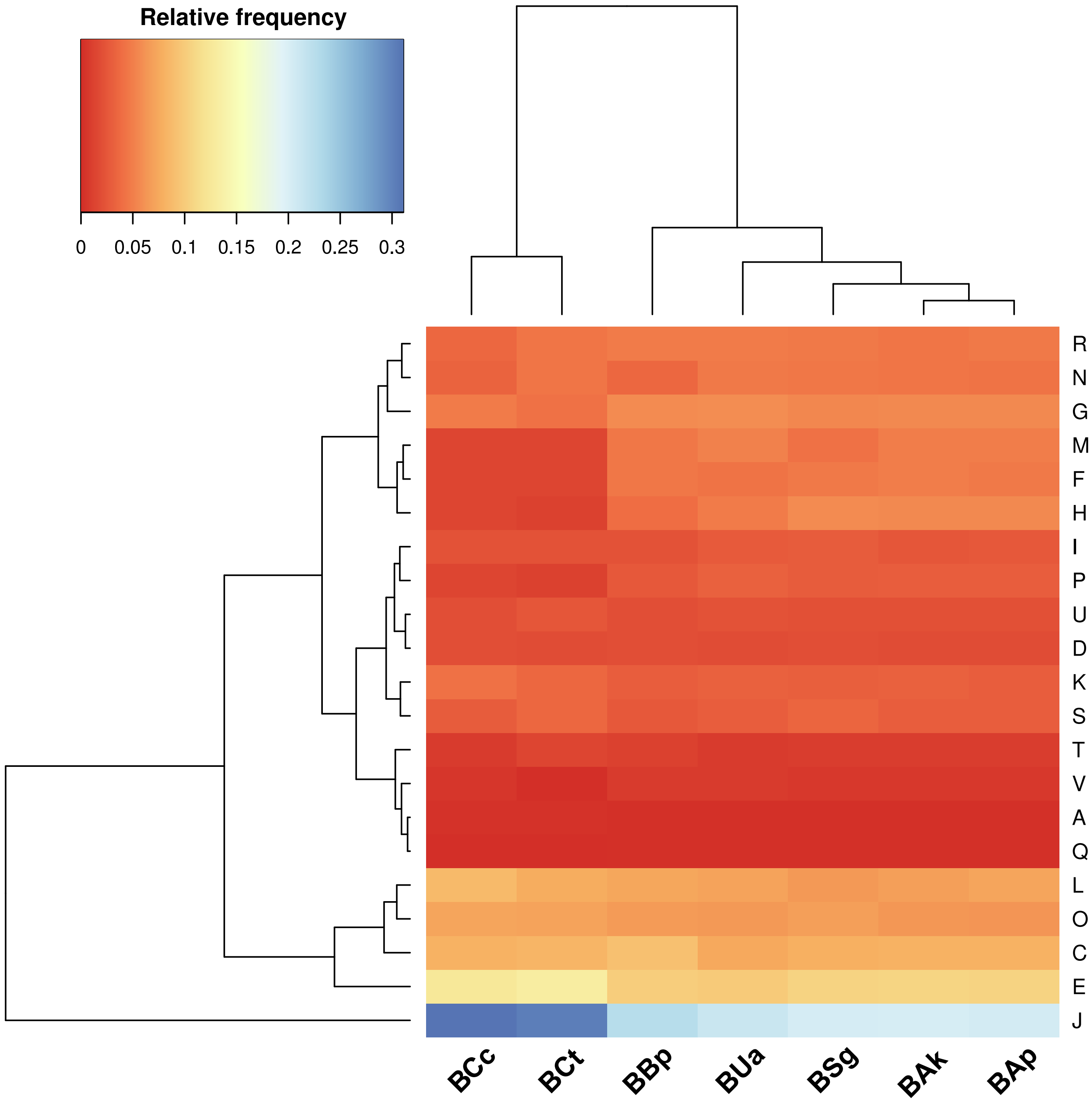


**Fig. S8. Functional profiles of *B. aphidicola* strains sequenced to date**

Heat map showing the the two-way clustering of COG profiles from the selected *B. aphidicola* strains using relative abundance. On the right side of the heat map, one-letter COG assignments for each row are displayed. On the bottom of each column, abbreviations for each of the *B. aphidicola* strains are shown as in supplementary **table S1**.
