# Supplementary material for: Settling Down: The Genome of Serratia symbiotica from the Aphid Cinara tujafilina Zooms in on the Process of Accommodation to a Cooperative Intracellular Life
Source: Genome Biol Evol. 2014 Jun 19;6(7):1683–98. doi: 10.1093/gbe/evu133 (PMC4122931; doi:10.1093/gbe/evu133)
Supplement: Supplementary Data [file supp_evu133_suppl_data.zip › suppl_file_4.pdf]

Supplementary Materials and Methods

Fluorescent In-Situ Hybridization

C. tujafilina insects were collected from the same location and tree as the ones used for sequencing, and embryos were extracted using tweezers in absolute ethanol and directly transferred into modified Carnoy's fixative (6 chloroform : 3 absolute ethanol : 1 glacial acetic acid) and left overnight (Koga et al. 2009).

Fixed embryos were then washed with absolute ethanol and transferred into a 6% solution of H2 O2 diluted in absolute ethanol and left for two hours. Hybridization was performed overnight at room temperature in standard hybridization buffer (20 mM Tris-HCl [pH 8.0], 0.9 M NaCl, 0.01% SDS, 30% formamide) and then washed (20 mM Tris-HCl [pH 8.0], 5mM EDTA, 0.1 M NaCl, 0.01% SDS) before slide preparation. Embryos were viewed under an Olympus FV1000 confocal microscope. EUB388- FAM (5'-FAM-GCTGCCTCCCGTAGGAGT-3') probe was used to mark eubacteria. We designed two probes targeting *B. aphidicola* and *S. symbiotica* 16S RNA molecules based on (Gómez-Valero et al. 2004): BCt-Cy5 (5'-Cy5- CCCGTTTGCCGCTTGCCGTCA-3') and SCt-DY-405 (5'-DY-405-CCGCCGCTCGTCACCCAGA-3'). These were tested for matches using TestProbe against the SILVA database (allowing one mismatch), finding them to be specific for the intended bacteria in *C. tujafilina*. RNase digestion and no-probe control experiments were done on embryos to confirm the specificity of the detection. All manipulations were performed at room temperature.

References

Gómez-Valero L et al. 2004. Coexistence of Wolbachia with Buchnera aphidicola and a secondary symbiont in the aphid Cinara cedri. J. Bacteriol. 186:6626–33. doi: 10.1128/JB.186.19.6626-6633.2004.

Koga R, Tsuchida T, Fukatsu T. 2009. Quenching autofluorescence of insect tissues for in situ detection of endosymbionts. Appl. Entomol. Zool. 44:281–291. doi: 10.1303/aez.2009.281.
